# GC-EI-MS identification data of neutral sugars of polysaccharides extracted from *Zizyphus lotus* fruit

**DOI:** 10.1016/j.dib.2018.01.085

**Published:** 2018-02-10

**Authors:** Khaoula Mkadmini Hammi, Majdi Hammami, Christophe Rihouey, Didier Le Cerf, Riadh Ksouri, Hatem Majdoub

**Affiliations:** a Laboratoire des Plantes Aromatiques et Médicinales (LPAM), Centre de Biotechnologie de Borj- Cédria, BP 901, 2050 Hammam-lif, Tunisia; b Laboratoire des substances bioactives (LSBA), Centre de Biotechnologie de Borj- Cédria, BP 901, 2050 Hammam-lif, Tunisia; c Normendie Université, Laboratoire de Polymères Biopolymères Surfaces(PBS), UMR 6270& FR3038CNRS, Université de Rouen, 76821 Mon Saint Aignan, France; d Université de Monastir, Laboratoire des Interfaces et des Matériaux Avancés (LIMA), Faculté des Sciences de Monastir, Bd. de l’environnement, 5019 Monastir, Tunisia

**Keywords:** Trimethylsilyl, Derivatization, GC–MS, Neutral sugar

## Abstract

Gas chromatography coupled to mass spectrometer (GC–MS) was used to identify and to quantify neutral sugars that constitute the water soluble polysaccharides from *Zizyphus lotus* fruit. The trimethylsilyl (TMS) method was successfully used for derivatization of the monosaccharides units of extracted polysaccharides that were released by hydrolysis method. Sugars were identified based on their retention times compared with those of standards and the NIST MS Spectral Library. All sugars were quantified in TIC (Total Ion Current) mode using calibration curves. Data is related to “Optimization extraction of polysaccharide from Tunisian *Zizyphus lotus* fruit by response surface methodology: Composition and antioxidant activity” (Mkadmini Hammi et al., 2016) [1].

**Specifications table**

**Table:** 

*Subject area*	*Chemistry*
*More specific subject area*	*Analytical chemistry*
*Type of data*	*Figure*
*How data was acquired*	*Gas chromatography coupled to mass spectrometer*
*Data format*	*Analyzed*
*Experimental factors*	*Lyophilized polysaccharides (ZLP) was hydrolyzed by trifluoroacetic acid (2M,70°C,2 h)*
*Experimental features*	*The released monosaccharides were converted to their trimethylsilyl (TMS) derivatives by adding 100 µL of dry pyridine and 100 µL of N,O-bis(trimethylsilyl)-trifluoroacetamide (BSTFA).*
*Data source location*	*n/a*
*Data accessibility*	*Data is within this article*

**Value of the data**

• The data can be used to identify neutral sugars of polysaccharides by GC–MS.
• Method and data provide information for acidic hydrolysis conditions of polysaccharides.
• The data provide information for trimethylsilyl (TMS) derivatization conditions of all neutral sugars for GC–MS analysis.

## 1 Data

**Fig. 1:**
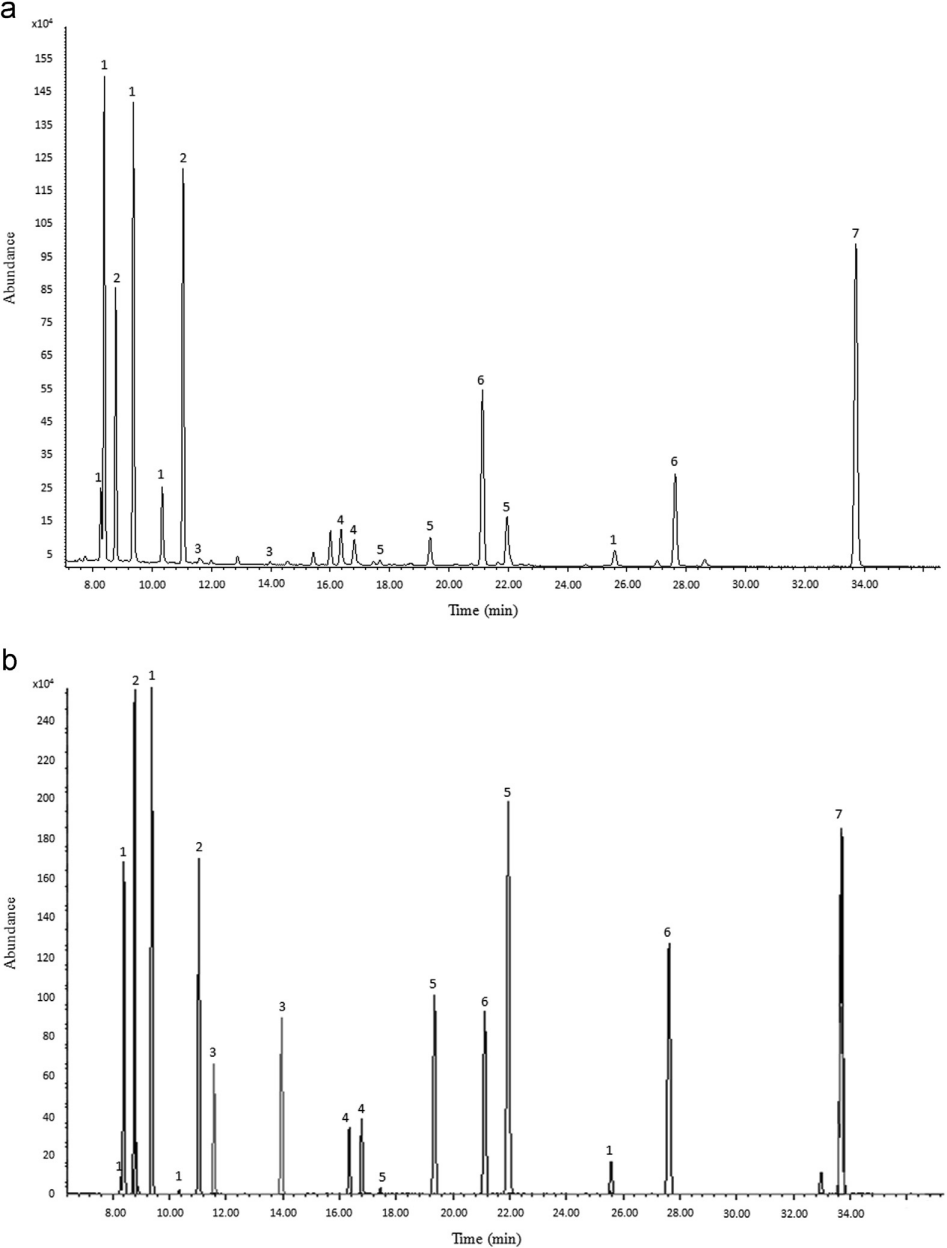
GC-EI-MS total ion current profiles of the trimethylsilylated sugars: a) ZLP; b) Standards mixture.

This data shows the GC-EI-MS total ion current profiles of the trimethylsilylated sugars obtained from ZLP and those of standards which injected under the same chromatographic conditions (Fig. 1).

## 2 Experimental design, materials and methods

0.45 mg of lyophilized ZLP was hydrolyzed by 1 mL of 2 M trifluoroacetic acid at 70 °C for 2 h in a sealed tube in nitrogen atmosphere. Then, 100 µL of D-myo-inositol (Internal standard) at a concentration of 1800 µg/mL in deionized water was added to the hydrolysate. The released monosaccharides were converted to their trimethylsilyl (TMS) derivatives by adding 100 µL of dry pyridine and 100 µL of N,O-bis(trimethylsilyl)-trifluoroacetamide (BSTFA) to the dried sample. The residue was dissolved in dichloromethane (100 µL) and 1 µL of silylated sample was injected to gas chromatography coupled to mass spectrometer analysis (Agilent technologies, 5975C inert MSD with its Triple-Axis Detector, Germany) [1].
