# Supplementary material for: GC-EI-MS identification data of neutral sugars of polysaccharides extracted from Zizyphus lotus fruit
Source: Data Brief. 2018 Feb 10;18:680–3. doi: 10.1016/j.dib.2018.01.085 (PMC5995744; doi:10.1016/j.dib.2018.01.085)
Supplement: Supplementary file 1 — Supplementary material [file mmc1.doc]

I certify that there is no conflict of interest about this Brief in Data concerning the paper :

Optimization extraction of polysaccharide from Tunisian *Zizyphus lotus* fruit by response surface methodology: Composition and antioxidant activity**.**

Mkadmini Hammi, K., Hammami, M., Rihouey, C., Le Cerf, D., Ksouri, R., & Majdoub, H. (2016).

*Food Chemistry*, *212*, 476-484.

(FOODCHEM-D-16-01383).

Khaoula MKADMINI HAMMI

Tunis, 18/17/2016
